# Supplementary material for: A phase I dose-finding design with incorporation of historical information and adaptive shrinking boundaries
Source: PLoS One. 2020 Aug 27;15(8):e0237254. doi: 10.1371/journal.pone.0237254 (PMC7451661; doi:10.1371/journal.pone.0237254)
Supplement: Attachment — Submitted filename: Response-v1.pdf [file pone.0237254.s002.pdf]

Response to the comments on manuscript PONE-D-20-07779 entitled "A Phase I Dose-finding Design with Adaptive Shrinking Boundaries and Incorporation of Historical Information – Extensions of the Bayesian optimal interval (BOIN) design "

We thank the editor and reviewer for their constructive comments. Based on the comments, we substantially revised our manuscript. In what follows, we copy your comments in italic and provide our point-by-point response.

Response to Editor's Comments

1. A rebuttal letter that responds to each point raised by the academic editor and reviewer(s). You should upload this letter as a separate file labeled 'Response to Reviewers'

Response: Done.

2. A marked-up copy of your manuscript that highlights changes made to the original version. You should upload this as a separate file labeled 'Revised Manuscript with Track Changes'.

Response: Done.

3. An unmarked version of your revised paper without tracked changes. You should upload this as a separate file labeled 'Manuscript'.

Response: Done.

4. We note that you have provided funding information that is not currently declared in your Funding Statement. However, funding information should not appear in the Acknowledgments section or other areas of your manuscript. We will only publish funding information present in the Funding Statement section of the online submission form. Please remove any funding-related text from the manuscript and let us know how you would like to update your Funding Statement. Currently, your Funding Statement reads as follows: 'The funders had no role in study design, data collection and analysis, decision to publish, or preparation of the manuscript.'

Response: Done.

5. Please amend either the title on the online submission form (via Edit Submission) or the title in the manuscript so that they are identical.

Response: *Done.*

6. Please amend either the abstract on the online submission form (via Edit Submission) or the abstract in the manuscript so that they are identical.

Response: *Done.*

7. Please ensure that you refer to Figure 4 in your text as, if accepted, production will need this reference to link the reader to the figure.

Response: *Done.*

8. Please thoroughly revise your manuscript for English grammar and spelling. It would be useful to have a native English speaker review the manuscript before it is resubmitted.

Response: *We've invited professional language editor to proof the language for the revised manuscript.*

Response to Reviewer 1's Comments

Major comments

1. Bayesian optimal interval design (BOIN) is a recently developed model-assisted method for phase I dose-finding trials. This design has been accepted as a popular design in this field due to its simplicity and desirable operating characteristics. In this paper, the authors extend the original BOIN design from two aspects: incorporate the prior knowledge and relax the fixed decision boundaries. Both of these two extensions are important since the 1st one could possibly improve the performance of the phase I trial if relevant prior information can be used and the 2nd is an interesting theoretical exploration of the original BOIN design. For the 1st extension, the authors proposed an automatic algorithm for its pre-specification of the parameter setups, which makes the proposed method feasibly to be used in real practices. The simulating conclusions are expected, which demonstrates the proposed algorithm works well empirically.

For the 2nd extension, though the results are not so exciting, that is, only when sample size are large, the proposed aBOIN design performs better than the original version, this exploration is still worthy from my perspective since it literally gives a solidified support for robustness of the original BOIN design.

My only concern is that it would be better that the paper can be edited by a professional language editor since there are still grammatic and language inappropriateness.

Response: *Thanks for your comments! For the language, we have invited professional language expert to edit the manuscript.*
